# Exploring perceived barriers, facilitators, and team roles in addressing prescribing cascades in primary care teams: Insights from interprofessional focus groups

**DOI:** 10.1371/journal.pone.0333829

**Published:** 2025-10-22

**Authors:** Sameera Toenjes, M. Christine Rodriguez, Sara J.T. Guilcher, Colleen Metge, Barbara J. Farrell, Lianne Jeffs, Lisa M. McCarthy

**Affiliations:** 1 Leslie Dan Faculty of Pharmacy, University of Toronto, Toronto, Canada; 2 Women’s College Hospital, Toronto, Canada; 3 Department of Family and Community Medicine, Temerty Faculty of Medicine, University of Toronto, Toronto, Canada; 4 Institute of Health Policy, Management and Evaluation, University of Toronto, Toronto, Canada; 5 Department of Community Health Sciences, Max Rady College of Medicine, University of Manitoba, Winnipeg, Canada; 6 Bruyère Health Research Institute, Ottawa, Canada; 7 Department of Family Medicine, University of Ottawa, Ottawa, Canada; 8 Science of Care Institute and Lunenfeld-Tanenbaum Research Institute, Sinai Health, Toronto, Canada; 9 School of Pharmacy, University of Waterloo, Waterloo, Canada; 10 Institute for Better Health, Trillium Health Partners, Mississauga, Canada; Universita degli Studi di Milano, ITALY

## Abstract

**Background:**

Prescribing cascades are common contributors to medication-related harm. Primary care clinicians who practice in interprofessional teams may be well positioned to address cascades in practice; although, the factors that influence their ability to do so in this particular setting are largely unknown. Our study aimed to explore perceived barriers and facilitators that influence the identification, investigation, and management of prescribing cascades as experienced by primary care teams and each profession’s role in addressing cascades.

**Methods:**

Physicians, nurse practitioners, and pharmacists practicing in primary care teams in Ontario, Canada participated in a series of intra-professional and interprofessional focus groups. The focus groups explored clinician perspectives about factors that influence their ability to address cascades. Discussion and analyses were guided by the Theoretical Domains Framework and the Behaviour Change Wheel.

**Results:**

Sixteen clinicians participated in four intra-professional and one interprofessional focus groups. Thematic analysis resulted in two main themes. First, multiple factors influenced primary care teams’ ability to address prescribing cascades. Cascades were considered complex in terms of the processes required to identify, investigate, and manage them. Second, the role each profession was able or willing to play varied based on their capability, opportunity, and motivation. Nurse practitioners and physicians felt best equipped to prevent cascades within a patient visit, while pharmacists endorsed being able and willing to address existing cascades.

**Conclusion:**

The ability to address prescribing cascades is influenced by multiple factors and varied across professions within interprofessional primary care teams. The study findings provide additional information necessary for the design of future interventions to assist clinicians with addressing cascades. Future research should engage patients, caregivers, and community pharmacists to further explore their roles in addressing prescribing cascades.

## Introduction

Prescribing cascades are increasingly recognized sources of potentially inappropriate prescribing and medication-related harm [1–5]. They occur when a new medication is started to treat the side effect of another medication [6,7], often when new signs or symptoms are attributed to a new medical condition rather than a medication’s side effect [6,7]. In cases where the harms outweigh the benefits of continuing both medications, a prescribing cascade can be thought of as problematic or inappropriate [8]. In addition to the harm related to their contributions to polypharmacy broadly, prescribing cascades have been associated with harms including hip fractures [9], hospital visits and admissions [10], and decreased quality of life [11].

A small number of studies exploring how and why prescribing cascades occur have illustrated the complexity with identifying, investigating, and managing them [4,5,12]. Current knowledge stems from four qualitative studies [4,5,13,14] and one cross-sectional survey study [15] conducted in Canada [4,5], Ireland [14,15], and the United States [13]. Two of the qualitative studies [4,5] involved interviews with physicians, pharmacists, patients, and their caregivers drawn from a geriatric day hospital [4,5], long-term care homes [5], and through a self-referral stream [5]. The third qualitative study examined educating patients with Alzheimer’s disease and their caregivers [13], while the fourth explored the views of many interest holders, i.e., patients, carers, care providers, professional organizations and policymakers, about prescribing cascades as part of polypharmacy [14]. Lastly, a cross-sectional survey study was conducted with community pharmacists [15]. These studies found that clinicians and patients face multiple barriers in addressing prescribing cascades, including difficulty identifying them and understanding whether a cascade occurred, unclear accountability, and lack of agreement on how to address cascades. Indeed, some clinicians, policymakers and the public consider prescribing cascades a ‘necessary evil’ that are part of the reality of managing the benefits of medication therapy [14].

To support the development of theory-informed interventions to address prescribing cascades, McCarthy et al [12] conducted a secondary analysis of the Canadian qualitative studies, which involved multiple care settings, using the Behaviour Change Wheel approach. The research team developed a multi-component behaviour, Ask-Investigate-Deprescribe (A-I-D), to describe points within the medication use cycle at which interventions are needed [12]. First, to prevent or identify cascades, clinicians need to determine if a patient’s sign or symptom can be caused by one or more of their medications (Ask). Second, to confirm a possible prescribing cascade, clinicians need to review the sequence of prescribing events and reasons for use of medications (Investigate). Third, if appropriate, deprescribing the medications (i.e., reducing doses or stopping medications) involved in the cascade can be undertaken to manage the cascade (Deprescribe). Importantly, investigation and management through deprescribing are often linked and iterative [12]. The authors also identified several potential intervention components that could be considered for addressing prescribing cascades across the health system broadly. To proceed with the next steps of intervention development, i.e., prioritize among the many potential intervention components in partnership with intended intervention participants, our team recognized the importance of carefully selecting a favourable contextual environment for intervention development and testing. Identification of a feasible setting is important for all intervention development but was particularly important for the case of prescribing cascades given the complexity of barriers reported [4,5,13,15] and the perceived inevitably of prescribing cascades by some parties [14]. Across prior studies, a key facilitator identified was care within an interprofessional team [4,5].

Primary care settings are considered the touchpoint for the integration of care in many health systems across the world [16,17]. In Canada, primary care prescribers write nearly 85% of all prescriptions [18]. Many models of primary care delivery exist in Canada; however, an increasing number of primary care prescribers seek to practice within interprofessional teams [19,20]. These teams often involve family physicians, nurse practitioners, and pharmacists in medication prescribing and management decisions. Primary care interprofessional teams were purposefully selected for prescribing cascade intervention development rather than individually practicing prescribers because individually practicing prescribers, e.g., family physicians, were expected to have little capacity to address prescribing cascades in daily practice. Comparatively, interprofessional teams were thought to possibly having more collective capability and opportunity due to their collaborative structure. Importantly, as previously explained, while past literature has elucidated the factors related to addressing prescribing cascades in a variety of settings (e.g., geriatric day hospital, long-term care, across the continuum of care), the factors within interprofessional primary care teams specifically are unknown. Further, to date, no study has explored the roles within interprofessional primary care teams that different primary care clinicians felt were best suited to address prescribing cascades and at which point during the medication use cycle within their daily practice realities. As such, to address these gaps and support future intervention development, in this study, we aimed to explore primary care clinicians’ perceived capabilities, opportunities, and motivation to identify, investigate, and manage prescribing cascades, and the roles of different professions within interprofessional primary care teams for engaging in these behaviours [21].

## Materials and methods

### Study design

This descriptive qualitative study used intra-professional and interprofessional focus groups to explore participants’ perspectives regarding the barriers and facilitators clinicians face in addressing prescribing cascades in primary care teams and their roles in identifying, investigating, and managing cascades in this setting. A post-positivist orientation guided study design [22]. Post-positivism can be argued to lie between positivist and constructivist world views. The post-positivist paradigm asserts that there is a single true reality, similar to positivism, however, there is an acknowledgement that humans cannot be objective given individual values and beliefs. With this subjectivity, individuals can only approximate true reality through research, but individuals never see true reality completely clearly [22]. As such, the research team’s unique values and assumptions are part of the research, and therefore, cannot be separated or removed from the data. The CORE-Q criteria were applied to guide study reporting [23].

### Researcher characteristics

The research team was composed of health professionals (four pharmacists, a physiotherapist, and a nurse) and researchers (three PhDs, one MSc, and one MSc student) with experience in qualitative research, behavioural science, and implementation science. All members were cis-gendered women. Two of the pharmacists had provided patient care in interprofessional primary care teams and all the health professionals had practiced within at least one interprofessional team in a variety of care settings (e.g., ambulatory clinics, hospitals).

### Eligibility, sampling and recruitment

Eligible participants included physicians, nurse practitioners, and pharmacists licensed under their respective colleges in Ontario, Canada who provided patient care in a primary care interprofessional team environment (i.e., team with two or more different professions) [24]. Participants’ licensing credentials were verified by the research team using publicly available regulatory database searches. English-language fluency was required, and those practicing in rural settings were prioritized for inclusion. Additionally, due to COVID-19 limitations on in-person gatherings at the time of data collection, participants needed stable internet access and access to and comfort with using Zoom^TM^ for the virtual sessions. There were no explicit exclusion criteria.

Both convenience and purposive sampling approaches were used to recruit primary care clinicians from diverse professions and practice settings. Recruitment approaches included targeted email, social media (Twitter/X, Facebook), and word of mouth. Study investigators developed a comprehensive list of invitees from their personal networks who were invited to participate by email. Relevant organizations were also contacted and asked to share information about the study via email and online. To recruit participants from rural areas, the team directly contacted rural Ontario family health teams through email to their publicly posted email accounts. Recruitment took place March 1 to April 30, 2022.

Written, informed consent was obtained from each participant prior to each focus group. The study was approved by the University of Toronto Research Ethics Board (#00042135).

### Data collection

Participant demographics (gender, age, years in practice, postal code for site of practice) were collected using a questionnaire administered with Qualtrics^TM^. At the time of study design, prior research demonstrated that ‘prescribing cascades’ were not readily recognized and well understood by many clinicians [4,5]. As such, to stimulate discussion in the focus groups, participants were given a one-page summary about prior research examining prescribing cascades for review prior to the focus group discussion. This was followed by the series of intra-professional and interprofessional focus groups, held in March and April 2022.

Focus groups (as opposed to individual interviews) were selected for data collection for a few reasons. First, they allowed us to explore the group’s thoughts on addressing cascades and provided the opportunity for clinicians to discuss differing opinions and build upon each other’s views in an open discussion [25]. This was particularly important for the inter-professional focus group as it allowed for confirmation of findings across the different professions and exploration of how primary care team members could work collaboratively to address prescribing cascades. Second, focus groups allowed us to have a larger sample size in a shorter time frame than could be accomplished with individual interviews. Finally, the virtual format allowed clinicians from diverse regions to efficiently share ideas and learn from each other [26].

All focus groups were held over Zoom^TM^ with discussions facilitated by ST (MSc student, PharmD) and LM (MSc, PharmD). Participants were aware of the facilitators’ professions as a pharmacist and role as an MSc student/ supervisor. An additional team member attended to provide technological support. Each virtual focus group (90–120 minutes per session) included up to five participants. A series of intra-professional focus groups (i.e., participants all within the same profession) were conducted: a group was held with physicians, another with nurse practitioners, and a final separate focus group was held with pharmacists. A pilot focus group was held to refine the questions in the discussion guide (S1 File. Appendix 3). Each intra-professional focus group began with a short presentation that provided an overview of prescribing cascades (drawn from the precirculated one page summary). Following the rapid analysis of the intra-professional focus groups (described next), an interprofessional focus group (i.e., participants from different professions) was convened to confirm findings across the different professions and explore how interprofessional teams could work collaboratively to address cascades. Physician, nurse practitioner, and pharmacist participants from the prior focus group sessions who consented to be re-contacted were invited to participate. This allowed the participants to provide their perspectives at multiple points in the data collection process. To stimulate discussion, the interprofessional focus group began with a presentation of findings from the rapid analysis of the intraprofessional focus groups.

Overall, participant risk with taking part in the focus groups was anticipated to be low as participants were invited to share their perspectives with no personally sensitive information discussed. The low but potential psychological or emotional risks pertaining to feelings of distress or anxiety in discussing individual views and experiences in a focus group with colleagues and researchers were acknowledged in study information materials and at the outset of the focus groups. Participants may have worried that the information shared during a focus group may have resulted in negative consequences both personally and professionally. To help mitigate this, expectations of privacy and respect within the focus group were emphasized before, during, and after the session and participants were encouraged to share only as much information as they were comfortable disclosing. Additionally, participants could take a break or withdraw at any time from a focus group discussion. There may have been a concern about privacy or the use of personal identifiable information within publications or shared data. To alleviate this, information that was considered identifiable by colleagues was removed when results were pooled and shared within the team or published. The discussion guide was developed using the results from the rapid analysis (S1 File. Appendix 4). During the focus group, findings from the rapid analysis were shared with the participants and participants’ reflections were collected.

### Data analysis

Code saturation was used to guide the number of focus groups [23,25]. Discussion and analysis were guided by the Theoretical Domains Framework (TDF) [27,28] and the BCW [21]. The BCW, a behavioural science-based approach for designing interventions, was used as it provides an approach for exploring mechanisms that can influence behaviour [21,29]. At the centre of the BCW is the Capability, Opportunity, Motivation, Behaviour (COM-B) Model, which explains that these three factors underlie behaviour [21]. The theory posits that to accomplish behavioural change, strategies need to target one or more of these factors. The 14 domains of the TDF connect to the COM-B model [21]. The TDF and BCW (i.e., COM-B model) were used to guide data collection, analyze the data, and organize the results.

A rapid analysis was conducted between the intra-professional and interprofessional focus group to allow for presentation of data to the interprofessional focus group. The approach was modeled on recent literature using this technique [30–33]. A draft summary table was created by ST, and reviewed and modified by LM. Inductive codes were created with short descriptors. Summary items described key perceived barriers and facilitators to prescribing cascades and what participants saw as opportunities for their role in addressing cascades. This was shared with the entire research team for feedback and was revised based on their input.

After the interprofessional focus group, the entire dataset was analyzed using an approach that incorporated features of codebook thematic analysis by Braun and Clarke to identify patterns and themes [34] and the DEPICT model for collaborative analysis within the team [35]. Inductive codes from the rapid analysis were used as a starting point, and the Theoretical Domains Framework and GUIDE-IT domains were used as sensitizing frameworks for the creation of the updated codebook. Four team members (ST, LM, CM, BF) were involved in coding the dataset using Microsoft Word and NVivo (S1 File. Appendix 5). Themes were derived from the data through regular team meetings and during two full team half-day retreats. Audit trails were kept and reviewed.

## Results

**Fig 1:**
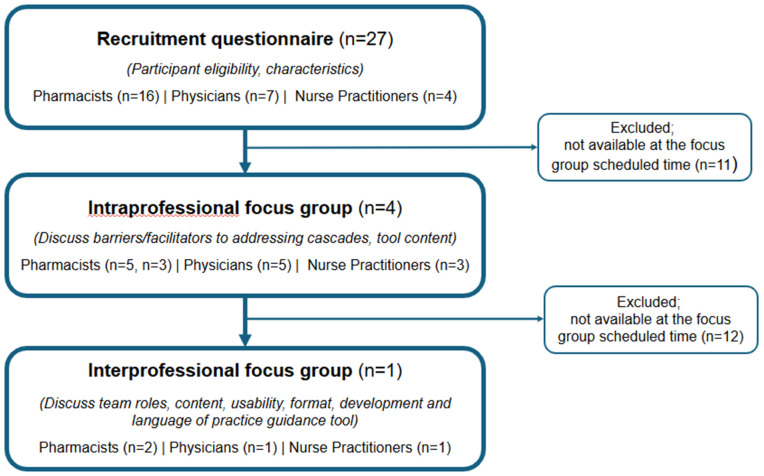
Participant flow diagram.

Sixteen clinicians participated in four intra-professional and one interprofessional focus groups. For the intraprofessional focus groups, one group was held with physicians, another with nurses and two were held with pharmacists because not all questions in the discussion guide were covered in the first pharmacist group. Fig 1 presents the flow of participants through the study. Most participants identified as women (75%) and practiced in an urban setting (94%). There were diverse representations of ages and years in practice across participants (S1 File. Appendix 1).

Two themes and several subthemes were identified and are summarized in Table 1.

**Table 1:** Study Themes.

Theme	Subthemes
1. Multiple factors influenced primary care teams’ ability to address prescribing cascades. Prescribing cascades were considered complex in terms of the processes required to identify, investigate, and manage them.	A. Primary care team clinicians endorsed varying accountability for addressing cascades depending on the clinical context of the specific patient case. B. Complexity related to the identification of cascades was compounded by challenges related to uncertain medication histories. C. Clinician and patient expectations for prescribing medications added to the complexity of investigating and managing cascades.
2. Professional role influenced capability, opportunity, and motivation to address cascades.	A. Nurse practitioners and physicians reported having limited capability and opportunity, affecting their motivation, to address prescribing cascades. These professions saw themselves as preventing cascades (i.e., by identifying that a sign or symptom could be a side effect before prescribing a new medication to treat it). B. Pharmacists endorsed having the capability, opportunity, and motivation to address cascades. Pharmacists saw themselves as able to address existing prescribing cascades during medication reviews.

### Theme 1: Multiple factors influence primary care teams’ ability to address prescribing cascades

**Fig 2:**
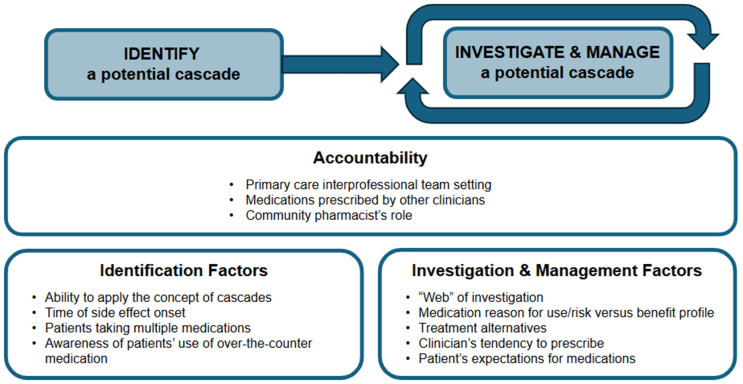
Factors influencing clinician’s ability to address cascades.

Participants reported several perceived barriers and facilitators that impact primary care teams’ ability to address prescribing cascades. These included accountability for addressing cascades broadly as well as other factors specific to identifying and then investigating and managing cascades (Fig 2).

#### Accountability factors.

Participants supported the notion that primary care teams are well-positioned to address cascades due to their longitudinal, trusting relationships with patients and the availability of support through interprofessional collaboration. The ability to consult interprofessional team members was perceived by participants as a benefit of this practice model. For example, both nurse practitioners and physicians reported benefits from consulting the pharmacist within their teams for managing drug therapy questions, including potentially addressing cascades.

The question of which professional(s) should be accountable for addressing cascades was raised by participants within all of the intra-professional groups. In this context, participants described accountability as having the means to “take ownership” of addressing cascades, “not only individually but also as members of a collective team” [36].

Overall, participants did not express clarity or consensus about who would ideally be accountable for addressing cascades. All participants were reluctant to accept accountability on behalf of their own profession due to pressures they faced including time limitations, high caseloads, etc. Participants across the focus groups were particularly reluctant to accept accountability for addressing cascades (i.e., changing or discontinuing medications) if another clinician initiated the cascade.

“Even working with the family physicians in my team, if there’s been, you know, something started by a specialist or a previous family doctor or something like that, there’s always so much hesitation and inertia around making changes.” (RPh75, interprofessional focus group)

Hesitation was also reported at times to be driven by patient concerns:

“She [the patient] didn’t want to make any changes, especially since it was the specialist who told her, it is okay to take it. So, I asked her to maybe try but you know. So, what I ended up doing was advise her to, you know, maybe follow up with her specialist to get some more clarification.” (RPh762, intra-professional focus group)

The suggestion that community pharmacists are professionals who could provide additional support to address cascades was raised by participants in all focus groups. This finding is particularly interesting because community pharmacists were not study participants nor were their roles raised or probed for by facilitators during focus groups.

#### Identification factors.

Participants reported that multiple factors influenced their ability to identify cascades. Overall, the concept of cascades was seen as complex. Participants reported having varying knowledge and understanding of the concept of cascades; some had difficulty applying the concept of cascades to examples in practice during the focus groups. Participants also discussed difficulties identifying cascades in situations where there is a delay between medication initiation and the onset of side effect(s).

The complexity of cascades was further complicated by unclear patient medication histories. Participants felt this lack of clarity was related to limited awareness of patients’ use of over-the-counter medications and instances where symptoms could be attributed to more than one medication or chronic condition.

“She [the patient] had very severe gastroparesis and she was on so many medications was certainly contributing to sedation and nausea anyway, but she had seen many, many specialists and I was really thinking that deprescribing was going to be what was going to help her, but it was just so hard to try and tangle out who it was who started it, what was on first, what was actually helping, and I didn’t get very far.” (MD650, intra-professional focus group)

#### Investigation and management factors.

Participants discussed various factors added to the complexity of investigating and managing cascades. This complexity was exacerbated by provider and patient expectations that favour prescribing (versus deprescribing) medications. Specifically, pharmacists spoke about the complexity of investigating cascades and the time needed to do so. Clinicians reported that when investigating prescribing cascades, the reason for a medication’s use and its risk versus benefit profile were often unclear and fluctuated over time. A lack of treatment alternatives was also considered a perceived barrier to managing cascades through deprescribing.

“And the only thing that she could tolerate was this venlafaxine. But as a side effect, it gave her high blood pressure. So, I had, I couldn’t switch her off of anything else. There was nothing left. It was just like we’d given her absolutely everything. And so, I had [to] start her on something for her blood pressure.” (NP747, intra-professional focus group)

During discussions about using deprescribing as a management strategy for prescribing cascades, a physician supported that they practice within a “culture of prescribing” (MD736, intra-professional focus group). A “culture of prescribing” refers to the tendency of clinicians to treat conditions by prescribing medications rather than considering other treatment options or deprescribing [4,5,13–15]. Participants also described sometimes feeling pressured by patients to continue prescribing medications rather than deprescribing them.

### Theme 2: Professional role influenced capability, opportunity, and motivation to address cascades

When considering potential roles for each profession in addressing cascades, broadly, we found that capability, opportunity, and motivation to address prescribing cascades varied by profession. Participants described three opportunities to best address cascades within a patient’s medication use process: (1) prevention (of a cascade before prescribing a second medication), (2) identification (of existing cascades), and (3) resolution (investigation and management of potential cascades). Table 2 provides an overview of potential roles for team members to intervene on prescribing cascades. S1 File – Appendix 7 provides representative quotations of participants’ reported capability, opportunity, and motivation to address prescribing cascades in practice.

**Table 2:** Potential Roles for Team Members at Points in a Patient’s Medication Use Process.

	Prevent	Detect	Manage
**Role**	Identify to prevent a potential cascade at the point of prescribing a new medication to avoid prescribing a second medication	Identify to detect a potential cascade during a medication review	Investigate and manage to resolve a potential cascade through actions such as deprescribing
**Example**	Clinician identifies that amlodipine may be causing peripheral edema and decides against prescribing furosemide	Clinician identifies that amlodipine and furosemide may be a potential cascade when both are present on a patient’s medication list	Clinician investigates the prescribing history for amlodipine and furosemide and if a cascade is likely, assesses appropriateness to determine whether management strategies such as deprescribing can be tried
**Professions who expressed interest to accept this role**	Nurse Practitioner Physician	Pharmacist	Physician Nurse Practitioner* Pharmacist**

*For nurse practitioners, if patients are rostered to them.

**Pharmacists reported they could provide guidance to nurse practitioners or physicians on how to deprescribe.

#### Physicians and nurse practitioners.

Given their central role in prescribing within primary care teams, both physicians and nurse practitioners felt potentially capable of preventing cascades, i.e., identifying potential cascades occurring at the point of prescribing; however, they described limited psychological capability to address cascades. They explained they were not formally taught to consider prescribing cascades in their assessments as part of their education. Due to this limited education, nurse practitioners viewed cascades as a “silent problem” (NP110, intra-professional focus group).

Nurse practitioners and physicians both described short patient visit times and the balancing of competing priorities as perceived physical opportunity barriers to addressing cascades. After reviewing the potential harms of cascades and discussing a sample case in the focus group, a physician reported:

“I might find myself too busy to do all that [understanding whether a patient’s furosemide is part of a prescribing cascade] and maybe add yet something else to sort of decrease the amount of urine he’s producing whether it’s night or day. I might dehydrate him more during the daytime, so he doesn’t have to pee at nighttime or something. I don’t know.” (MD717, intra-professional focus group)

Both nurse practitioners and physicians reported they would only be able to address cascades if they could do so during patient visits given they conduct patient assessments and develop management plans within, and not outside, a patient visit. Nurse practitioners and pharmacists suggested that pharmacists may be better positioned to address existing cascades as they typically have longer patient visits. If nurse practitioners and physicians needed to address existing cascades themselves, they reported the ideal time to do this would be when patients are stable and suggested periodic health exams as a potential time to do this with their rostered patients.

Nurse practitioners who did not have patients rostered to them personally described additional physical and social opportunity challenges when addressing cascades. These challenges were related to both physical opportunity (i.e., the absence of historical relationships with patients, difficulty providing longitudinal follow-up) and social opportunity (i.e., concerns about “playing in their [patient’s primary care provider’s] sandbox” (NP163, intra-professional focus group)). As a result, they reported hesitation in managing prescribing cascades through deprescribing.

Varying levels of reflective and automatic motivation for addressing cascades were also reported. After participating in the intra-professional focus group, a physician described how he was motivated to address prescribing cascades, but this motivation may only be temporary. Participants shared ideas related to automatic motivation that could influence their engagement with cascades in the future, e.g., participation in audit and feedback programs, legislated structured medication reviews, financial incentives.

#### Pharmacists.

Pharmacists described having the psychological capability – both the knowledge (i.e., medication expertise) and skills (i.e., clinical thought process) – needed to identify and investigate existing prescribing cascades. They reported that their education (i.e., considering medication-related causes of symptoms) prepared them to identify cascades.

Conversely, pharmacists variably endorsed their psychological capability to manage cascades (i.e., develop plans to deprescribe and monitor the impact of medication changes). One pharmacist described using deprescribing as both an investigation and management strategy to understand a medication’s risk versus benefit profile:

“I went back, and I was saying, okay, well, I understand that you know, you’re on this medication, you know, it’s causing this side effect. But let’s talk about like, what the actual benefit of pregabalin is to you [...] And like, maybe it is worth taking it off, maybe both of these medications, the spironolactone and the pregabalin are doing me more harm than good altogether. And so, like, slowly, by slowly, we’ve started working at chipping away the pregabalin, and just seeing if her pain changes or not.” (RPh75, intra-professional focus group).

In contrast, other pharmacists described uncertainty about the optimal approach to managing prescribing cascades. Some expressed uncertainty about where to start when thinking through possible approaches for managing cascades when discussing the sample case, while others felt unclear about the best strategy to manage the cascade (i.e., Should both medications be changed simultaneously? Should one medication be changed at a time? If so, which medication should one change first?). These questions suggest that some pharmacists may require additional guidance on how to deprescribe cascades.

With respect to opportunity, pharmacists reported having the social and physical opportunity to address existing cascades given their role in conducting medication reviews and their ability to take the time to do the necessary investigative and management work (including deprescribing). Pharmacists reported limited opportunities for preventing cascades as they are often not present during patient visits when prescriptions are written. Instead, pharmacists reported multiple experiences with addressing existing cascades. Pharmacists also endorsed the observation made by physicians and nurse practitioners that appointments for patient medication reviews are typically longer than for other professions, providing sufficient physical opportunity to address existing cascades.

Pharmacists’ reflective and automatic motivation seemed to be related to their professional identity and successful experiences with deprescribing. Several pharmacists saw addressing cascades as part of their professional role, i.e., as medication resources or experts on their teams. Pharmacists also shared multiple successful experiences where deprescribing improved their patients’ quality of life. This led to professional optimism, and, therefore, reflective motivation, toward addressing medication-related harm generally. These positive experiences with investigating and managing cascades increased pharmacists’ motivation to address prescribing cascades for future patients.

## Discussion

This study is the first to explore factors that influence the ability of Canadian interprofessional primary care teams to address prescribing cascades, including examining the roles different primary care clinicians perceive as best suited within these interprofessional teams to address prescribing cascades. We found that their ability to address prescribing cascades is influenced by multiple factors and that the capability, opportunity, and motivation to address cascades varied by profession.

With respect to self-identified roles for addressing cascades, nurse practitioners and physicians reported both limited capability and opportunity, affecting their motivation to address cascades. These two professions saw their role to be within preventing cascades, i.e., by identifying that a sign/symptom could be a side effect before prescribing a new medication to treat, rather than in addressing existing prescribing cascades. Pharmacists endorsed having the capability, opportunity, and motivation to address cascades. They saw themselves as able to address existing prescribing cascades during medication reviews.

Prior studies have explored factors influencing clinicians and the public’s ability to address cascades across several care settings [4,5,13–15], including long-term care homes and geriatric day hospitals [4,5]. Further, since deprescribing is intertwined with investigating and managing prescribing cascades, studies exploring factors that influence deprescribing in primary care settings are also of relevance [37–44]. S1 File – Appendix 8 provides a fulsome summary of how factors identified in the present study align with those reported for prescribing cascades in other settings [4,5,13] and deprescribing [37–44]. Briefly, many of the factors reported by our study participants affecting their ability to address prescribing cascades are consistent with those reported in prior studies focused on cascades [4,5,13]. These included clinicians not considering medication side effects during their assessments, a general lack of information about patient’s medications (i.e., benefit, history, and reason for use), and the complexity of the iterative, non-linear process of investigation and management of cascades [4,5].

Our findings also validate what has been reported in previous studies focused on deprescribing in primary care settings. Specifically, clinicians lack of familiarity with identifying deprescribing opportunities and how to deprescribe [39,43,44], patient [40], and provider [37,41] fear of deprescribing, and poor communication across care settings [42] have been reported in previous knowledge syntheses and qualitative studies. Specific influences on community pharmacists’ involvement in deprescribing, which were not explored specifically in this study, have also been explored [38,45].

As discussed, previous work has described perceived barriers and facilitators related to addressing prescribing cascades in a variety of settings (e.g., geriatric day hospital, long-term care, across the continuum of care); however, the perceived barriers and facilitators related to addressing prescribing cascades within interprofessional primary care teams specifically were unknown. Our study adds to this body of literature by providing insight into clinician capability, opportunity, and motivation to address prescribing cascades in the interprofessional primary care team context. This information is essential for those wishing to design initiatives to address cascades focused on this care setting. Further, the results from the present study also validated that many of the barriers and facilitators described in the past literature on deprescribing and prescribing cascades also apply to addressing prescribing cascades in interprofessional primary care teams.

Limitations of the present study include the recruitment strategy, the use of focus groups for data collection, and variability of integration of interprofessional primary care teams into some health systems. Study recruitment occurred mostly through emails and social media which may have limited the diversity of our sample. This may be less of a concern for healthcare professionals (versus the general public) as they often engage in virtual care and other professional activities online [46]. Focus groups often face criticism due to concerns with internal power dynamics [25] that may limit honest conversation [47]. This was important to consider in the interprofessional focus group given the known power dynamics among different professions in healthcare [47]. To mitigate this, the facilitator for all focus groups emphasized that the discussion was meant to be open and non-judgmental. While interprofessional primary care teams are increasingly common in some countries, such as Canada and the United Kingdom, there are varying states of implementation of the model in countries across the world. Given this, our results may not be fully transferrable to primary care practice globally.

While our recruitment strategy had limitations, it was also quite robust. We purposely recruited participants of varied ages, years of experience, and from both urban and rural practices sites. In addition, our data analysis approach adds to the rigor of our study. Data analysis included both inductive and deductive coding using both rapid [48–50] and thematic analysis [34]. The rapid analysis step allowed us to gather participant feedback on our interpretation of data from the intraprofessional focus groups and provided an inductive start to our codebook. The thematic analysis added the Theoretical Domains Framework domains and BCW as deductive sensitizing frameworks to allow for a more in-depth understanding of our dataset. This step-wise approach allowed us to view the data from multiple lenses. Team members, with diverse experiences in qualitative research, implementation science and as licensed healthcare providers, participated in coding as well as development of themes, consistent with the DEPICT model [35]. Team members were familiar with the dataset and actively discussed their reflections on the results, adding rich interpretations to our analysis.

For future research, our study findings about the capability, opportunity, and motivation of each profession can be mapped to corresponding intervention types, policy categories, and behaviour change techniques using the BCW approach to develop interventions. In addition, while this study focused on the perspective of clinicians in primary care teams, the direct involvement of patients, caregivers, community pharmacists, and other interested groups whose roles are relevant to addressing prescribing cascades remain underexplored, both in the existing literature and in the present study.

## Conclusions

This research has provided unique insights into the complexity of identifying, investigating and managing prescribing cascades within primary care settings. The roles that interprofessional primary care team members were able or willing to play varied based on their capability, opportunity, and motivation. Nurse practitioners and physicians felt best equipped to *prevent* cascades during a patient visit, while pharmacists endorsed being able and willing to *address* existing cascades.

## Supporting information

S1 File Study Appendices. Includes Appendices 1–8.
(DOCX)
