# Supplementary material for: Exploring perceived barriers, facilitators, and team roles in addressing prescribing cascades in primary care teams: Insights from interprofessional focus groups
Source: PLoS One. 2025 Oct 22;20(10):e0333829. doi: 10.1371/journal.pone.0333829 (PMC12543151; doi:10.1371/journal.pone.0333829)
Supplement: S1 File — Includes Appendices 1–8. (DOCX) [file pone.0333829.s001.docx]

Exploring barriers, facilitators, and team roles in addressing prescribing cascades in primary care teams: Insights from interprofessional focus groups – **Supplementary Files**

**Study Appendices**:

Appendix 1: Study Participant Characteristics

Appendix 2: Mapping Codebook Thematic Analysis and DEPICT to the Steps of Analysis

Appendix 3: Intra-professional Focus Group Discussion Guide

Appendix 4: Interprofessional Focus Group Questions

Appendix 5: Coding Tree

Appendix 6: Representative Quotations on Factors Influencing Clinicians’ Ability to Address Cascades

Appendix 7: Representative quotations for capability, opportunity, motivation to address prescribing cascades in practice

Appendix 8: Factors Influencing Clinician’s Ability to Address Cascades in Relation to Past Literature

#

# SUPPLEMENTARY APPENDICES

| Appendix 1: Study Participant Characteristics | | |
| --- | --- | --- |
| Category | Divisions | N (%) |
| Gender | Women  Men | 12 (75)  4 (25) |
| Age range (years) | 25 to 35  36 to 45  46 to 55  56 to 65 | 9 (56)  2 (12.5)  3 (19)  2 (12.5) |
| Profession | Physicians  Nurse Practitioners  Pharmacists | 5 (31)  3 (19)  8 (50) |
| Years in practice  (years) | Under 5  5 to 15  16 to 30  Over 30 | 6 (38)  4 (25)  5 (31)  1 (6) |
| Area of practice  (based on postal code) | Urban  Rural | 15 (94)  1 (6) |

| Appendix 2: Mapping Codebook Thematic Analysis and DEPICT to the Steps of Analysis | | |
| --- | --- | --- |
| Step of Analysis | Codebook Thematic Analysis | DEPICT (1) |
| 1. Creating the codebook  2. Coding an intra-professional transcript  3. Coding an interprofessional transcript  4. Coding remaining transcripts | Phases 1 to 3 (i.e., familiarizing, generating, and searching for themes) | Dynamic Reading  Engaged codebook development  Participatory coding |
|  |  |  |
|  |  |  |
|  |  |  |
| 5. Research team retreat to review codes, quotes, potential themes  6. Creation and review of code summary statements grouped by potential theme  7. Synthesis of results | Phases 3 to 5 (i.e., searching for, reviewing, defining and naming themes) | Inclusive reviewing and summarizing of categories  Collaborative analyzing  Translating |
|  |  |  |
|  |  |  |

## Appendix 3: Intra-professional Focus Group Discussion Guide

Introduction

1. (Round table) Everyone takes 1 min to introduce yourself and tell us about your patient care practice (e.g., the size of your care team, how many patients you look after).
2. Tell us about your experience with a prescribing cascade. Share a situation when you have spotted a possible prescribing cascade and what happened next (i.e., how that was handled?).

Probes:

- Anything you did that worked well?
- Are there any tools you use that are helpful?

1. After going through what we have learned about prescribing cascades (their prevalence, harms, and proposed steps to tackle them in practice) and the barriers and facilitators to deprescribing, does all of this resonate with you when thinking about prescribing cascades in your practice?

Probes:

- If no, what’s missing?
- Are there any ‘aha’ or lightbulb moments? Any muddy points?

1. Is the evidence we have about prescribing cascades sufficient to convince you to invest in addressing them?

Probes:

- Is there anything additional that you would need to know about prescribing cascades before you would be willing to invest in addressing them?
- Is it enough to clearly state the quality of evidence we have now?
- I’m hearing that people want outcomes data, and it’s in progress - people are starting to look at that - is that a non-starter? Should we not go down the path of a tool until we have that?

1. What do you see as your role in addressing prescribing cascades in practice?

Probes:

- What do you see as your role in addressing prescribing cascades in practice?
- Do you see yourself collaborating with anyone? What role do they play?
- What are the barriers to or what would help perform this role?
- Relationship with the team where it works - are there sometimes it doesn’t work?

1. In what ways could a practice guidance tool or tools be helpful for you to be able to address prescribing cascades in practice?

Probes:

- This is a great discussion! To be a little more specific, what would make it more or less helpful?
- If need to prompt discussion, think back to Esa’s medication list and potential cascades. What do you think you’d need to address prescribing cascades?

1. What should be included in a practice guidance tool?

Probes:

- What guidance would you need to make it possible to address prescribing cascades in practice?
- What content would you want to be included to make it applicable/relevant to you and your patients?
- What are the puzzle pieces/building blocks?

If silence

- An easy example is that it may be helpful to have a list of common cascades

If still no discussion

- Would something like a list of questions to help you investigate be useful?
- Or a decision tree to help solve if it is problematic or not and what to do next?
- What questions would be useful to ask?

1. Would you prefer general guidance like a thought process or specific directions on how to approach each individual prescribing cascade?

Probes:

- Are the general questions or a blueprint enough? (answering it in the hypothetical)
- If it goes down the path of specific directions… since there have been so many targets identified, what are your thoughts about how we would prioritize them?

1. How would you want this tool to work within your day-to-day activities?

Probe:

- When and how do you see yourself using it?
- Do you see different healthcare providers covering different steps?
- How long? How flexible?

1. Are there certain organizations that you would look for an endorsement from?
2. What’s the best way to involve primary care clinicians?
3. Are there any additional comments or thoughts related to today’s discussion - the idea of developing a practice guidance tool for prescribing cascades - that you wanted to mention?

## Appendix 4: Interprofessional Focus Group Questions

Introductions (10 min - including presentation)

1. In alphabetical order, maybe we’ll do a quick 1 min overview of who you are and your practice.
2. We’re here to discuss prescribing cascades so my first question is - who feels up for refreshing the group about what a cascade is?
3. (if time) Since we last saw you, has anyone seen any cascades in practice? Will anyone share an experience… Thanks X, let’s keep going.
   1. (may have to dig or clarify if someone gives you something that isn’t a cascade i.e., don’t end this part if the example is confusing)
   2. [if example not a prescribing cascade] Sounds like a great example of medication-related harm, just to make sure we’re all on the same page prescribing cascades are …

Review of Profession’s Roles for Prescribing Cascades (10 min)

1. How does this fit for you? Does this align with what you would envision as possible roles for the different professionals?

- Probe: Is there anything we are misinterpreting or missing here?
- Probe: How often in your practices are you reviewing medications?

Review Practice Guidance Tools from Intra-Professional Focus Group (5 min)

Content (25 min)

1. Which of these tools do you see as most useful or helpful and why?
   1. Make sure everyone responds
   2. Probe: What do you like about it?
   3. Probe: How would you use the most helpful one in your own practice?
2. Do you see different professions needing different tools?
   1. If only suggest identification or management tools: What would you see as useful to pair this tool with? What next?
   2. Probe: If yes, which profession would start with to make a tool?
3. (if time) At what point would you like to involve patients? (i.e., with conversation starters or patient decision aids)

Practice Environment (20 min)

1. If each profession has different roles, we have been debating which profession would be the most impactful place to start. Who do you see as needing this support the most and being able to use it?
   1. Probe: At what point in the prescribing/patient care process does it make sense to start?
   2. If they can't answer from a team, what about their own profession? Who do you think can make the most impact (recognizing more NPs/MDs on an FHT team) but also has the bandwidth and appetite?
      1. Note: goal may be towards prevention
2. What might encourage or get in the way of the uptake of the tool in practice? (if it’s a specific tool)

Development (10 min)

1. How would it be best to involve primary clinicians in the development process?
2. Would it be useful to pair with any existing tools (STOPP/START or Beer’s list, TaperMD)? Do any of you use these tools?
3. (if time) Are there any other organizations (besides ChoosingWisely & professional organizations) you would value endorsement from?

Usability (10 min) - less of a priority if run short on time

1. Given this debate about whether to put this on an external platform (such as an app, pocket guide, or webpage) vs. an internal source (integrated into an electronic medical record system). What would be your preference?
2. Would you prefer a tool that can be used as part of a patient visit in real-time or outside of the visit?

Wrap-Up (5 min)

1. Does anyone have any questions about what’s been said so far?
2. Does anyone have anything else to add before we wrap up?
3. (If time) Round table for reflections.

## Appendix 5: Coding Tree

| **Code Name** | **Description** |
| --- | --- |
| Theoretical Domains Framework | Captures codes related to the research question, “What barriers and facilitators do Ontario primary care clinicians experience with identifying, investigating and managing prescribing cascades in their practices?”- using the Theoretical Domains Framework as a sensitizing framework. There are 14 main codes related to the theoretical domains framework (listed below). |
| Behavioural Regulation (around RxC) | Captures codes about managing or changing objectively observed or measured actions in relation to addressing prescribing cascades  Constructs: Self-monitoring, Breaking habit, Action planning |
| Identification and monitoring of side effects | Discussions about behaviour regulation in distinguishing between side effects and new symptoms  E.g., determining whether nausea is a side effect of a current medication or a symptom of a new/changing condition |
| Investigation & Treatment of side effects | Discussions about behaviour regulation around investigating symptoms / side effects to determine their cause and assessing, monitoring and treating those side effects. |
| Beliefs about Capabilities (to address RxC) | Discussions about the acceptance of the truth, reality, or validity in the ability to tackle prescribing cascades in practice  Constructs: Self-confidence, Perceived competence, Self-efficacy, Perceived behavioural control, Beliefs, Self-esteem, Empowerment, Professional confidence |
| Professional confidence | Discussion about professional confidence in the ability to tackle prescribing cascades in practice  -can include self-confidence if speaking in regard to their profession |
| Beliefs about Consequences (when addressing RxC) | Discussions about the acceptance of the truth, reality, or validity about outcomes of a tackling prescribing cascades in a given situation  Constructs: Beliefs, Outcome expectancies, Characteristics of outcome expectancies, Anticipated regret, Consequents |
| Emotion (towards RxC) | Captures codes related to emotions, or complex reaction pattern, involving experiential, behavioural and psychological elements, by which the individual attempts to deal with a personally significant matter or event  Constructs: Fear, Anxiety, Affect, Stress, Depression, Positive / negative affect, Burn-out |
| Provider’s emotions about changing medications | Descriptions of hesitancy, reluctance, anxiety, fear on the part of the provider to make changes to medications that a patient is currently taking |
| Patient’s emotions about changing medications | Descriptions of hesitancy, reluctance, anxiety, fear on the part of the patient to make changes to medications that they are currently taking |
| Environmental Context and Resources (for RxC) | Captures codes related to any circumstance of a person’s situation or environment that discourages or encourages the development of skills & abilities, independence, social competence, & adaptive behaviour to tackle prescribing cascades in practice  Constructs: Environmental stressors, Resources / material resources, Organizational culture / climate, Salient events / critical incidents, Person x environment interaction, Barriers and facilitators |
| Role of community RPh | Discussions on role of community pharmacists in helping tackle prescribing cascades |
| Influence of COVID-19 | Discussions on the influence of the COVID-19 pandemic in tackling prescribing cascades |
| Frequent use of episodic / acute care | Discussions on patient’s frequent use of episodic / acute care and how that affects provider’s ability to tackle prescribing cascades |
| Team collaboration | Discussions on instances / examples of collaboration / communication processes between providers within the same team about medications  E.g., wholesome approach, access to interprofessional team, access to local notes, medical directives for RPh to change doses, many prescribers |
| Collaboration with specialists | Discussions in instances / examples of collaboration / communication processes between primary care providers and specialists about medications  E.g., lack of care coordination, care in silos, lack of shared communication / records, many prescribers |
| Having rostered vs. un-rostered patients | Discussions of how having rostered patients or having un-rostered patients and how that influences a providers’ ability to address prescribing cascades |
| Influence of EMR | Discussions relating to the electronic medical record & its role in addressing prescribing cascades |
| Tools used to identify RxC | Discussions about existing tools providers use to identify prescribing cascades in current practice  E.g., (from data) drug information resources (UpToDate), guidance on inappropriate prescribing (Beer’s criteria), devices to get a better patient history (Freestyle Libre) |
| Unclear medication indication | Discussions of how the indication for a medication can be unclear in practice |
| Goals (in RxC) | Descriptions of goal setting - I.e., mental representations of outcomes or end states that an individual wants to achieve - regarding prescribing cascades  Constructs: Goals (distal / proximal), Goal priority, Goal / target setting, Goals (autonomous / controlled), Action planning, Implementation intention |
| Intentions (towards RxC) | Descriptions of intentions - i.e., a conscious decision - to address prescribing cascades in practice  Constructs: Stability of intentions, Stages of change model |
| Knowledge (of RxC) | Captures codes related to knowledge - i.e., an awareness of the existence - of prescribing cascade, both regarding a specific case/cascade as well as more general understanding of medication side effects  Constructs: Knowledge, Procedural knowledge, Knowledge of task environment |
| Educational training | Descriptions of impact of educational training on providers’ understanding / knowledge / awareness of prescribing cascades and relevant medication literacy |
| Familiarity with RxC | Descriptions of providers’ familiarity or unfamiliarity with the terminology/concept of prescribing cascades |
| Medication side effects | Descriptions of providers’ knowledge and / or awareness of potential side effects of a given medication |
| OTC medications | Descriptions of providers’ knowledge regarding over the counter medications including their potential side effects, status as drugs, etc. |
| Memory, Attention, and Decision Processes (regarding RxC) | Captures codes related to the ability to retain information, focus selectively on aspects of the environment and choose between two or more alternatives in relation to prescribing cascades  Constructs: Memory, Attention, Attention control, Decision making, Cognitive overload / tiredness |
| Decision processes for RxC | Discussions of providers’ decision-making processes for prescribing cascades |
| Visit priority | Discussions of how prescribing cascades are or are not prioritized during patient’s visit  E.g., if a patient is stable (vs. acutely ill or undergoing a transition in care) and when to address them |
| Planned follow up | Discussions of ability to follow up with patient’s prescribing cascade(s) |
| Consideration of treatment alternatives | Discussions of the consideration of treatment alternatives when considering deprescribing for prescribing cascades |
| Optimism towards RxC | Captures codes related to optimism - confidence that things will happen for the best or that the desired goals will be attained - when addressing prescribing cascades.  Constructs: Optimism, Pessimism, Unrealistic optimism, Identity |
| Optimism towards RxC’s | Discussions of provider’s optimism in tackling prescribing cascades |
| Pessimism towards RxC’s | Discussions of provider’s pessimism in tackling prescribing cascades |
| Reinforcement (for RxC) | Captures codes related to increasing the probability of a response by arranging a dependent relationship, or contingency, between the response and a given stimulus regarding addressing prescribing cascades  Constructs: Rewards (proximal / distal, valued / not valued, probable / improbable), Incentives, Punishment, Consequents, Reinforcement, Contingencies, Sanctions |
| Patient awareness / self-advocacy | Discussion of patient awareness of personal prescribing cascades and how they advocate for themselves |
| Past experiences tackling RxC’s | Discussion of provider’s past experiences with tackling prescribing cascades in practice |
| Skills (to address RxC) | Captures codes related to skills - I.e., an ability or proficiency acquired through practice - to tackle prescribing cascades  Constructs: Skills, Skills development, Competence, Ability, Interpersonal skills, Practice, Skill assessment |
| Ask or Identify RxC | Discussions if providers ask / assess if the sign or symptom can be caused by one or more of the drugs the patient is taking  Captures steps involved in identifying and confirming the presence of a prescribing cascade |
| Ask before adding new med (prevention) | Discussion of identifying RxC’s before adding a new medication - considering drug-related causes of signs and symptoms  In other words, the “asking” part of that behaviour, before prescribing a new medication to prevent a RxC |
| Ask during medication reviews (retrospective) | Discussion of identifying prescribing cascades during medication reviews to detect existing cascades - after second medication is prescribed, retrospective review |
| Investigate RxC | Discussions of the providers investigating sequence of events and reasons for medications related to a prescribing cascade |
| Understanding on-going benefit | Descriptions of challenges in determining whether a given medication remains beneficial in addressing a target symptom/condition/etc  -challenges described in understanding on-going benefit, or lack thereof, of a current medication, for example, a patient has been taking a given medication for many years and it is no longer clear what, if any, benefit they are deriving from it |
| Manage RxC | Descriptions of managing the prescribing cascades in practice |
| Barriers to Deprescribing | Descriptions of the barriers to deprescribing medications particularly in the context of a suspected/identified cascade  Deprescribing is defined as: A. To prevent a cascade or manage an existing one, you need to decrease, pause or STOP potentially causative drug(s) (drug A), monitor for adverse drug withdrawal events (ADWEs) B. Then, if cascade exists, decrease, pause or stop drug B and monitor for ADWEs |
| Patient preferences | Descriptions of provider's accounts of patient preferences regarding taking medications, i.e., their current or past medications; patient’s preference regarding taking medications in general; e.g., their preference for not taking medications at all or for seeking/using non-pharmaceutical treatment options, their preference for certain types of medication/treatment over others |
| Risk-benefit analysis | Discussions of experiences of weighing potential risks and benefits of maintaining a suspected / identified prescription cascade for a given patient |
| Social influences (around RxC) | Captures codes relating to those interpersonal processes that can cause individuals to change their thoughts, feelings or behaviours towards prescribing cascades  Constructs: Social pressure, social norms, Group conformity, social comparisons, Group norms, social support, Power, Intergroup conflict, Alienation, Group identity, Modeling |
| Patient Expectations | Discussion of patient expectations and how they influence the ability to tackle prescribing cascades  E.g., patient pressure to get Rx  (may be captured under patient’s emotions about changing medications) |
| Patient-Provider Relationship | Discussions of the relationships between patients and providers, particularly with regards to medication management and prescribing cascades  E.g., patient-provider trust |
| Social or Professional Role & Identity | Captures codes related to social or professional role & identity - I.e., coherent set of behaviours and displayed personal qualities of an individual in a social or work setting – in relation to prescribing cascades  Constructs: Professional identity, Professional role, social identity, Professional boundaries, Professional confidence, Group identity, Leadership, Organizational commitment |
| Nurse practitioner role & identity | Discussions on how nurse practitioners perceive their professional role & identity in tackling prescribing cascades |
| Pharmacist role & identity | Discussions on how pharmacists perceive their professional role & identity in tackling prescribing cascades |
| Physician role & identity | Discussions on how physicians perceive their professional role & identity in tackling prescribing cascades |
| GUIDE-M Domains | Contains all codes related to research question, “What do primary care teams need in terms of a practice guidance tool(s) to recognize, investigate and manage prescribing cascades?” using the GUIDE-M domains (content, development, format, language, and practice environment) as a sensitizing framework. |
| Utility of PGT | Descriptions related to the perceived UTILITY of having a practice guidance tool to assist with tackling prescribing cascades in practice  -answers question: “How could a practice guidance tool be useful to you?” |
| Content of PGT | Contains all codes related to the CONTENT of a potential practice guidance tool  E.g., information about specific context (e.g., goals & targets), information about medications (e.g., implicit vs. explicit tool), information about steps to take (e.g., analysis of risks vs. benefits of intervening) |
| Electronic alert or flag | Descriptions of a tool that provides an alert or flag when a new medication is prescribed that could be a part of a new potential prescribing cascade  -include data relating to alert fatigue |
| List of symptoms & drug-related causes | Descriptions of a tool that lists common presenting symptoms & potential drug-related causes.  E.g., tool for use with older adults to that lists drugs that may cause insomnia, edema, cough, or rash |
| Medication list analyser | Descriptions of a tool (e.g., app, website, or button within EMR) that analyzes a patient’s medication list and provide feedback about potential prescribing cascades |
| Tool to match conditions to medications | Descriptions of a tool that would allow a clinician to match medical conditions to medications as a way of identifying medications that may not have a clear purpose & may be part of a cascade |
| Stepwise algorithm for specific cascades | Descriptions of a tool that’s a stepwise algorithm for specific cascades with directions on what to do.  E.g., (from rapid analysis) idea of a tool with two arms to it - one that is the prevention arm - to prevent cascades at the point of prescribing that is proactive & more NP/MD focused; and one arm on how to manage existing ones that may be more reactive & RPh focused.  Examples of what to include within tool: flags for specialist referral, risks & benefits of addressing the cascade, directions on how to adjust medications, alternative medications to drug causing side effect, monitoring parameters, when to consider re-prescribing medications, etc. |
| General thought process for cascades | Descriptions of a tool that would be a general approach / thought process for how to manage any cascade  -include data on how general thought process may allow more room for clinical judgment based on patient specific circumstances |
| Patient conversation starter | Descriptions of a tool that would help patients bring up the idea of prescribing cascades with their provider |
| Patient decision aid | Descriptions of a tool that would be a patient decision aid to help patient’s weigh risks vs. benefits of deprescribing or continuing a cascade |
| Implicit vs. Explicit Tool | Discussions of whether the tool should be implicit (general guidance for any cascade) vs. explicit (guidance for specific cascades) |
| Prioritizing RxC | Discussions of how to consider prioritizing prescribing cascades (at a practice or individual-patient level) within a practice guidance tool  E.g., based on the impact of prescribing cascade on patient outcomes, severity of side effects, etc. |
| Development of PGT | Contains all codes related to the DEVELOPMENT of the practice guidance tool. Development includes discussion of trustworthiness, development process, developers, etc. |
| Development process for PGT | Descriptions of the suggested development process for the practice guidance tool  E.g., how to make the tool & consult stakeholders |
| Evidence synthesis in PGT | Descriptions of the specific context, evidence, and information for synthesis within a practice guidance tool |
| Quality of evidence in PGT | Descriptions of quality of evidence (e.g., need for RCTs, consensus-based approach, etc) suggested to create a practice guidance tool |
| Partnerships for creating PGT | Descriptions of the trustworthiness of the developers of the practice guidance tool.  E.g., (from data) organizations to partner with to increase its trustworthiness such as ChoosingWisely, professional organizations (e.g., NPAO) |
| Format of PGT | Contains all codes related to the FORMAT / layout of the practice guidance tool. Format includes format type (e.g., summaries vs. detailed descriptions), organization (e.g., charts vs. paragraphs), presentation (bullet points vs. sentences), platform (e.g., electronic vs. paper-based) |
| Platform for PGT | Descriptions of the type of platform to house the practice guidance tool  E.g., online platform, within electronic medical, paper-based |
| Format type for PGT | Descriptions of how the information should be presented within the practice guidance tool  E.g., detailed descriptions vs. simple summary, visuals (if any) |
| Organization of PGT | Descriptions relating to the organization of practice guidance tool  E.g., algorithm, good practice guideline, charts, lists, etc. |
| Models for PGT | Discussions of what to model this practice guidance tool after  E.g., (from data) CEP, TaperMD |
| Language of PGT | Discussions related to the LANGUAGE of the practice guidance tool.  E.g., Clarity, Specificity, Actionability, Sensibility |
| Practice Environment for PGT | Contains all codes related to the PRACTICE ENVIRONMENT for the practice guidance tool. In other words, how does the practice guidance tool fit into day-to-day activities? |
| Evolution of PGT | Discussions of how the practice guidance tool will evolve in practice  E.g. starting as an external tool used by pharmacists and evolving into a tool that is integrated into the EMR and used by all professions |
| Accessibility of PGT | Discussions about the accessibility of the tool  E.g., external app vs integrated into EMR; used within visit vs. outside of visit |
| Time / opportunity to tackle RxC | Discussions about the perceived time / opportunity to tackle prescribing cascades within the workday  (Summary from rapid analysis:  -for NP, may be during an acute episodic issue (if not rostered) / during a periodic health review (if rostered)  -for MD, potentially during a periodic health review / preventative health screening exam  -for RPh, may be during a complex medication review  -across all: QI project within the practice) |
| Usability of PGT | Descriptions related to the USABILITY of the practice guidance tool more.  E.g., Simplicity, Applicability, Updating, Ease of use, Consistency, Flexibility |
| Miscellaneous | Capture any interesting data related to the research question that does not fall within a specific code |

| S6 Appendix: Representative Quotations on Factors Influencing Clinicians’ Ability to Address Cascades | |
| --- | --- |
| Factor | Representative Quotation |
| Accountability | “I think it's [primary care teams] a really good place because it's often the place where the patient has the most history. So, it probably [would] be easiest to find out like when things were started and why.” (MD736, intra-professional focus group)  “The pharmacist within our family health team is absolutely excellent. […] And you will also find that in the family health team where we specifically refer for, you know, drug interactions, and maybe deprescribing and those kinds of things. It's extremely, extremely helpful.” (MD717, intra-professional focus group)  “Especially as their [community pharmacists] scopes have expanded so much with what they can bill OHIP [Ontario Health Insurance Plan] for now that there's probably a lot of untapped potential there for them to be the identifiers, which then takes some of the pressure off of us.” (NP163, intra-professional focus group) |
| Identification | “Because it was a long time the patient had been on the midodrine. So, I mean, you wouldn't even really think about it. But of course, it is one of the side effects of midodrine that eventually your blood pressure will go up.” (MD717, intra-professional focus group) |
| Investigation and Management | “I kind of was visualizing almost like a web because there's so many different interconnected reasons for what's going on […] Can I look at it [the medical conditions and medications] in a slightly different way? Have I missed an angle? [...] we're taught in a linear function in a linear manner [...] But then you get out you realize it's anything but linear.” (RPh747, intra-professional focus group)  “...and then chronic pain on its own is just totally difficult. Because, you know, someone could have a good week here and a bad week there. And it's really hard to know, you know, is the drug helpful or not.” (RPh75, intra-professional focus group)  “The culture of prescribing is very true. I feel like often the patient comes on a drug and you're like, oh, someone somewhere saw a good enough reason to start this person on a drug. And so even if you don't necessarily think it's clinically indicated, it's actually really difficult to deprescribe because you're like, am I gonna destabilize some well-controlled chronic condition and do more harm than good? And then if then it's kind of like, oh, then I have to like, try and find out who prescribed it. And as I try to contact whoever prescribed it, and sometimes that's not so easy.” (MD736, intra-professional focus group)  “There's also a lot of pressure from patients that they just want a medication to fix things. So, it's hard to have those discussions. And then I find when I do try to tackle deprescribing, it's often interpreted incorrectly by the patient, especially if they're older that they're thinking you're trying to tell them that they're not worthy or in the end of life, and that's not kind of what you're trying to say.” (NP163, intra-professional focus group) |

| Appendix 7: Representative quotations for capability, opportunity, motivation to address prescribing cascades in practice | |
| --- | --- |
| Physicians/ Nurse practitioners | Representative Quotations |
| Capability | “I don't think that we necessarily get a lot of formal training on it [deprescribing, prescribing cascades] in our medical education.” (MD903, intra-professional focus group)  “It is a silent problem for a lot of patients in just the terms that the patients and the clinicians themselves may not be fully aware of all these cascades and just like NP163 said, it's identifying them that is the big challenge.” (NP110, intra-professional focus group)  “If they're coming to see me for an acute episode, it could be swelling, like leg swelling or something and it's just like their one thing, what they're there for, and then analyze their medications to see if there's common ones that cause edema.” (NP110, interprofessional focus group) |
| Opportunity | “For the most part with these sorts of things, if we don't do it while patients [are] there, you're not going to do it.” (MD717, interprofessional focus group)  “The challenge I see with that is I feel like in clinic, primary care, you're often so pressed for time. […] a good opportunity to do it would be kind of like the annual periodic health reviews where you're trying to get go through everything, I think, like, then annually could trigger you to, to go through that with your roster.” (MD736, intra-professional focus group)  “But one of the big things I find problematic in my practice is that, like I said, I don't have patients rostered to me. So [...] you see them in once or twice, and you just can't make all those changes, because you're not their primary care practitioner, and you can make the suggestions, but it never gets followed through. Or yeah, you get that, ‘uh, well, I'll see what my doctor says before I make any changes.’ And it's very frustrating.” (NP110, intra-professional focus group) |
| Motivation | “...I mean, we all should do it [address prescribing cascades]. But we all should do so many other things, too, right? So, you know, you sit there, and you say, ‘Well, what's my priority today?’ [...] Prescription cascades are high in my mind for the last couple of weeks. You know, what's the next thing that sort of takes my mind away from prescription cascades?” (MD717, interprofessional focus group) |
| Pharmacists | Representative Quotations |
| Capability | “The way I've been trained, and the first thing that I think of is, are any of the drugs causing the symptoms?” (RPh75, intra-professional focus group).  “I went back, and I was saying, okay, well, I understand that you know, you're on this medication, you know, it's causing this side effect. But let's talk about like, what the actual benefit of pregabalin is to you [...] And like, maybe it is worth taking it off, maybe both of these medications, the spironolactone and the pregabalin are doing me more harm than good altogether. And so, like, slowly, by slowly, we've started working at chipping away the pregabalin, and just seeing if her pain changes or not.” (RPh75, intra-professional focus group).  “So, let's say, amlodipine and then furosemide. So, what that means is that you need to then switch amlodipine, first, to another antihypertensive, that doesn't cause it. In the meantime, you think you're thinking about, you know, should I continue with the furosemide or not? Should I change two meds at the same time?” (RPh762, interprofessional focus group) |
| Opportunity | “I feel that there are two arms to it. One is how to prevent it from happening, so stop new drug prescribing. And then a second arm would be like what to do when we have a patient in front of you. And how do you identify and reduce that. So, I feel that with the first one, how to prevent them they may target more for the prescribers [nurse practitioners and physicians] [...] the second arm would be more [...] for pharmacists when we're doing med reviews.” (RPh762, intra-professional focus group)  “We're [pharmacists in family health teams are] uniquely placed that, you know, I'm not seeing 35 patients in a day say like they are. So, if they, you know, something comes up around deprescribing or we don't know, I can take the time to do the deep dive through the chart and find the line and the through line of the story and actually come back and say, ‘This is why this, this, this and this.’” (RPh872, intra-professional focus group)  “I think most primary care pharmacist referral processes that will we have the luxury of getting the referral before the patient's right in front of us and being able to kind of sit and think with the information a little bit first. So, I don't think it necessarily needs to be, if you're thinking about specifically something that's targeting pharmacist audience, I don't think it needs to be something that can be done in visit. I think a lot of my work and thought process is usually done kind of when the patient isn’t like, right speaking with me on the phone, or like in person.” (RPh75, interprofessional focus group) |
| Motivation | “Those drug-induced side effects are kind of where we [pharmacists] can offer that other perspective that maybe other healthcare professionals don't think of first, it's kind of the first thing we [pharmacists] think of when we look at medication list and a problem that patient is having is oh, well, could any of their meds be causing it? And so, I think that kind of uniquely positions us both our expertise, and we have the time to be able to do these kind of deep dives.” (RPh747, intra-professional focus group)  “We were able to drastically you know, adjust his insulin doses. See where the patterns were. Improved that. That when I called, even I'm trying to think now, maybe a week later, his wife said, I got my old husband back. His energy, he's not napping during the day, he's out with these puttering about in the work shed. He's sleeping better. He's not having nightmares, he's not waking up...” (RPh872, intra-professional focus group) |

## Appendix 8: Factors Influencing Clinician’s Ability to Address Cascades in Relation to Past Literature

| 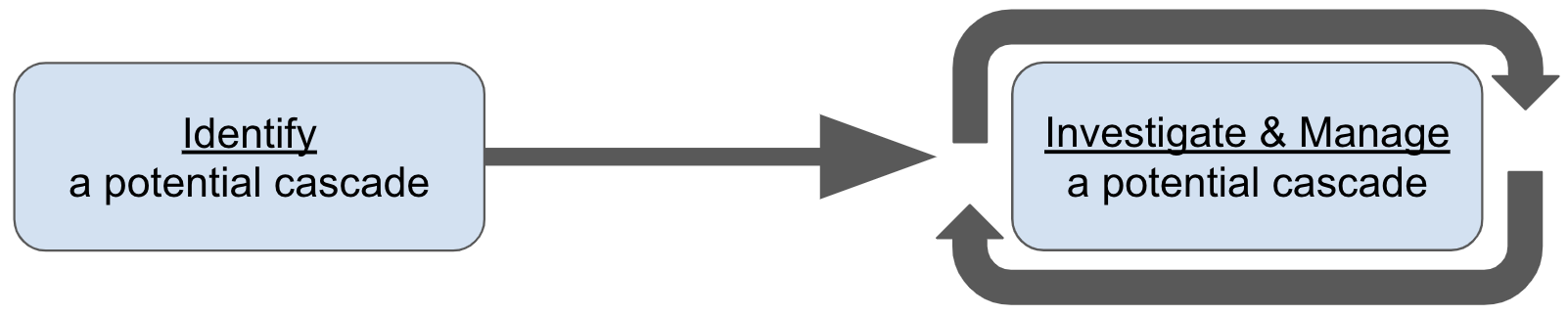 | |
| --- | --- |
|  |  |
| **Accountability**   - Primary care interprofessional team setting - Medications prescribed by other clinicians (2,3) - Community pharmacist’s role (4,5) | |
|  |  |
| **Identification Factors**   - Ability to apply the concept of cascades (2,3,6) - Time of side effect onset (2,3) - Patients taking multiple medications (2,3) - Awareness of patients’ use of over-the-counter medication (7,8) | **Investigation & Management Factors**   - “Web” of investigation (2,3,9) - Medication reason for use/risk versus benefit profile (2,3,9) - Treatment alternatives (10) - Clinician’s tendency to prescribe (11–13) - Patient’s expectations for medications (10,14) |
| Appendix H: Factors Influencing Clinician’s Ability to Address Cascades in Relation to Past Literature | |

# References

1. Flicker S, Nixon SA. The DEPICT model for participatory qualitative health promotion research analysis piloted in Canada, Zambia and South Africa. Health Promot Int. 2015 Sep 1;30(3):616–24.

2. Farrell BJ, Jeffs L, Irving H, McCarthy LM. Patient and provider perspectives on the development and resolution of prescribing cascades: a qualitative study. BMC Geriatr. 2020 Sep 25;20(1):368.

3. Farrell B, Galley E, Howell P, McCarthy L. “Kind of blurry”: Deciphering clues to prevent, investigate and manage prescribing cascades. PLOS ONE. 2022 Jun 28;

4. Korenvain C, MacKeigan LD, Dainty KN, Guilcher SJT, McCarthy LM. Exploring deprescribing opportunities for community pharmacists using the Behaviour Change Wheel. Res Soc Adm Pharm RSAP. 2020 Jan 31;

5. Farrell B, Clarkin C, Conklin J, Dolovich L, Irving H, McCarthy L, et al. Community pharmacists as catalysts for deprescribing: An exploratory study using quality improvement processes. Can Pharm J Rev Pharm Can. 2020 Jan 1;153(1):37–45.

6. Prescribing cascades in persons with Alzheimer’s disease: engaging patients, caregivers, and providers in a qualitative evaluation of print educational materials - Sarah Bloomstone, Kathryn Anzuoni, Noelle Cocoros, Jerry H. Gurwitz, Kevin Haynes, Vinit P. Nair, Richard Platt, Paula A. Rochon, Sonal Singh, Kathleen M. Mazor, 2020 [Internet]. [cited 2021 Apr 12]. Available from: https://journals-sagepub-com.myaccess.library.utoronto.ca/doi/full/10.1177/2042098620968310

7. Doherty AJ, Boland P, Reed J, Clegg AJ, Stephani AM, Williams NH, et al. Barriers and facilitators to deprescribing in primary care: a systematic review. BJGP Open. 2020 Aug;4(3):bjgpopen20X101096.

8. Gerlach N, Michiels-Corsten M, Viniol A, Schleef T, Junius-Walker U, Krause O, et al. Professional roles of general practitioners, community pharmacists and specialist providers in collaborative medication deprescribing - a qualitative study. BMC Fam Pract. 2020 Sep 4;21(1):183.

9. Jennings AA, Doherty AS, Clyne B, Boland F, Moriarty F, Fahey T, et al. Stakeholder perceptions of and attitudes towards problematic polypharmacy and prescribing cascades: a qualitative study. Age Ageing. 2024 Jun 1;53(6):afae116.

10. Reeve E, To J, Hendrix I, Shakib S, Roberts MS, Wiese MD. Patient barriers to and enablers of deprescribing: a systematic review. Drugs Aging. 2013 Oct;30(10):793–807.

11. Anderson K, Stowasser D, Freeman C, Scott I. Prescriber barriers and enablers to minimising potentially inappropriate medications in adults: a systematic review and thematic synthesis. BMJ Open. 2014 Dec 8;4(12):e006544.

12. Wallis KA, Andrews A, Henderson M. Swimming Against the Tide: Primary Care Physicians’ Views on Deprescribing in Everyday Practice. Ann Fam Med. 2017 Jul 1;15(4):341–6.

13. Anderson K, Foster M, Freeman C, Luetsch K, Scott I. Negotiating “Unmeasurable Harm and Benefit”: Perspectives of General Practitioners and Consultant Pharmacists on Deprescribing in the Primary Care Setting. Qual Health Res. 2017 Nov;27(13):1936–47.

14. Kennie-Kaulbach N, Cormier R, Kits O, Reeve E, Whelan AM, Martin-Misener R, et al. Influencers on deprescribing practice of primary healthcare providers in Nova Scotia: An examination using behavior change frameworks. Med Access Point Care. 2020 Jan 1;4:2399202620922507.
